# Early Visual Processing and Perception Processes in Object Discrimination Learning

**DOI:** 10.3389/fnins.2021.617824

**Published:** 2021-01-28

**Authors:** Matías Quiñones, David Gómez, Rodrigo Montefusco-Siegmund, María de la Luz Aylwin

**Affiliations:** ^1^Centro de Investigaciones Médicas, Universidad de Talca, Talca, Chile; ^2^Facultad de Educación, Universidad de O’Higgins, Rancagua, Chile; ^3^Instituto de Aparato Locomotor y Rehabilitación, Facultad de Medicina, Universidad Austral de Chile, Valdivia, Chile; ^4^Escuela de Medicina, Universidad de Talca, Talca, Chile; ^5^Programa de Investigación Asociativa (PIA) en Ciencias Cognitivas, Centro de Investigación en Ciencias Cognitivas, Universidad de Talca, Talca, Chile

**Keywords:** object discrimination learning, encoding time, perception, recognition memory, ROC

## Abstract

A brief image presentation is sufficient to discriminate and individuate objects of expertise. Although perceptual expertise is acquired through extensive practice that increases the resolution of representations and reduces the latency of image decoding and coarse and fine information extraction, it is not known how the stages of visual processing impact object discrimination learning (ODL). Here, we compared object discrimination with brief (100 ms) and long (1,000 ms) perceptual encoding times to test if the early and late visual processes are required for ODL. Moreover, we evaluated whether encoding time and discrimination practice shape perception and recognition memory processes during ODL. During practice of a sequential matching task with initially unfamiliar complex stimuli, we find greater discrimination with greater encoding times regardless of the extent of practice, suggesting that the fine information extraction during late visual processing is necessary for discrimination. Interestingly, the overall discrimination learning was similar for brief and long stimuli, suggesting that early stages of visual processing are sufficient for ODL. In addition, discrimination practice enhances *perceive* and *know* for brief and long stimuli and both processes are associated with performance, suggesting that early stage information extraction is sufficient for modulating the perceptual processes, likely reflecting an increase in the resolution of the representations and an early availability of information. Conversely, practice elicited an increase of *familiarity* which was not associated with discrimination sensitivity, revealing the acquisition of a general recognition memory. Finally, the *recall* is likely enhanced by practice and is associated with discrimination sensitivity for long encoding times, suggesting the engagement of recognition memory in a practice independent manner. These findings contribute to unveiling the function of early stages of visual processing in ODL, and provide evidence on the modulation of the perception and recognition memory processes during discrimination practice and its relationship with ODL and perceptual expertise acquisition.

## Introduction

Experts can quickly and correctly discriminate and individualize images from an expertise category, such as cytopathological images (Crowley et al., 2003; Evered et al., 2013), X-Rays (Boutis et al., 2010; Waite et al., 2019), and fingerprints (Searston and Tangen, 2017). The ability to discriminate complex visual images is acquired through extensive practice (Gibson, 1963; Goldstone, 1998; Fine and Jacobs, 2002; Seitz, 2017) or exposure in natural conditions (Tanaka, 2001; Shen et al., 2014; Saffran and Kirkham, 2018).

The experts’ great speed and accuracy with the behavioral responses (Gauthier et al., 1998; Rossion et al., 2001; Tanaka et al., 2005; Shen et al., 2014; Harel, 2016) are based on more efficient processing (Curby and Gauthier, 2010; Richler et al., 2011; Harel, 2016; Reeder et al., 2016) associated to a reduced stimulus processing time and an increase in the resolution of the stimuli representations. For stimulus processing time, early work showed that object categorization at a detailed or subordinate level (ex. breed) is slower compared to the basic level categorization (Grill-Spector and Kanwisher, 2005). Moreover, expertise and familiarity speeds up the categorization at the subordinate level to match the basic level and constraining stimulus duration, or encoding time, reduces performance, with a greater effect for less familiar objects (Grill-Spector and Kanwisher, 2005). Overall, this evidence is consistent with the coarse-to-fine information theory of visual processing and the expertise or familiarity reducing the time for fine information extraction (Curby and Gauthier, 2009). Summarizing, experts and novices differ in the encoding time required to discriminate and individuate objects.

Imaging and behavioral studies show that the visual processing of faces (Goffaux et al., 2011; Cichy et al., 2014; Besson et al., 2017; Dobs et al., 2019), for which humans are highly experts, follows a sequential path from coarse to fine information extraction. A similar processing sequence has been suggested for scenes (Musel et al., 2014; Loschky et al., 2015). More importantly, experience and familiarity with the stimuli improves the information available in the early stages of processing (Dobs et al., 2019), in agreement with the behavioral results (Curby and Gauthier, 2009). Specifically, face processing includes an image decoding stage that onsets about 46 ms and peaks around 103 ms, followed by gender and age information extraction and ends with the extraction of the identity information. Interestingly, the information about identity is small for unfamiliar faces, which likely prevents their discrimination. Thus, the visual processing in experts and novices differs substantially within the first 1,000 ms after stimulus onset due to the amount of information available for processing in the early stages. Yet, no studies have examined whether the early and late stages of visual processing are required for object discrimination learning (ODL) and experience acquisition. Moreover, expertise acquisition is also associated with an increase in the resolution of the representations at different levels of processing for visual (Scolari et al., 2008; de Beeck et al., 2019; Blauch et al., 2020) and auditory stimuli (Bao et al., 2004; Han et al., 2007; Bao, 2015). Yet, no studies have evaluated if the early and late stages of visual processing are required for the modulation of the resolution of representations during ODL.

In addition to the modulation of visual processing by expertise acquisition, repetitive stimuli exposure and discrimination leads to the acquisition of recognition memory for known (Nyhus and Curran, 2009; Herzmann and Curran, 2011) as well as for novel stimuli (Musen and Treisman, 1990; Schacter and Cooper, 1993). Moreover, the study time or stimulus duration determines the type of memory engaged in memory tasks (Martini and Maljkovic, 2009; Voss and Gonsalves, 2010) where a long stimulus (>1 s) promotes recognition memory while a brief stimulus (0.25 s) promotes priming. Surprisingly, expert cytologists and radiologists exhibit a much greater recognition memory for common objects compared to memory in the domain of expertise (Evans et al., 2011), suggesting that perceptual expertise for complex stimuli does not necessarily involve recognition memory. In summary, recognition memory is engaged during the repetitive viewing of stimuli and is modulated by stimulus duration, but it is still not clear whether it is required for perceptual expertise acquisition. To the best of our knowledge, no study has evaluated the perception and recognition memory processes during visual ODL and expertise acquisition.

Recognition memory and perception processes can be estimated from behavioral judgments according to the dual process theory of memory, in which recognition memory judgments are based on two independent processes: a “state” all or none threshold process *of recall* and a “strength” gradual signal detection process of *familiarity* (Mandler, 1980; Yonelinas, 2001). Likewise, the perception judgments are based on two independent processes; a “state” process of perceiving if two stimuli are the same, and a “strength” process of knowing if two stimuli are same (Aly and Yonelinas, 2012). The memory and perception processes have been estimated with the receiver operating characteristics (ROC) curves (Aly and Yonelinas, 2012; Dube and Rotello, 2012). Thus, using ROC curves, we can estimate the progression of perception and memory state and strength processes during discrimination practice and ODL.

Here, we evaluate the impact of encoding time and information extraction in the acquisition of ODL, by conducting an experiment with two tasks. The first is a sequential matching task in which we compared ODL with brief and long encoding times corresponding to the early and late stages of visual processing, respectively. In a second perceptual/recognition memory evaluation task, we estimated the progression of perceptual and recognition memory during ODL. Four groups of participants discriminated pairs of same or different exemplars of initially unfamiliar scrambled checkerboard-like images. According to the coarse to fine theory of expert visual processing, if ODL involves image decoding and coarse and fine information extraction we can anticipate that a long encoding time, that includes early and late stages of processing, will result in greater learning. Alternatively, if image decoding and early visual processing, involving early stages of processing are sufficient for discrimination learning, then brief and long encoding times will result in similar overall discrimination learning. Moreover, if perceptual processes are the main factors modulated during discrimination learning, then practice will promote “strength” and “state” processes of perception and they will associate with performance and ODL. Likewise, if recognition memory processes are engaged in discrimination learning, then practice will promote “strength” and “state” processes of memory and their association with performance and ODL.

## Materials and Methods

### Participants

Forty-three right-handed participants, mean age 21.4 (range 18–26) all male, with normal or corrected-to-normal vision, participated in this study in exchange for monetary compensation (approximately $40 US dollars). All participants, college or graduate students recruited through advertisements placed in the University of Talca, gave written informed consent before the behavioral measurements. The experiments were conducted in accordance with Protocol # 2015-091-MA, approved by the Ethical Committee of the University of Talca.

### Stimuli

**FIGURE 1:**
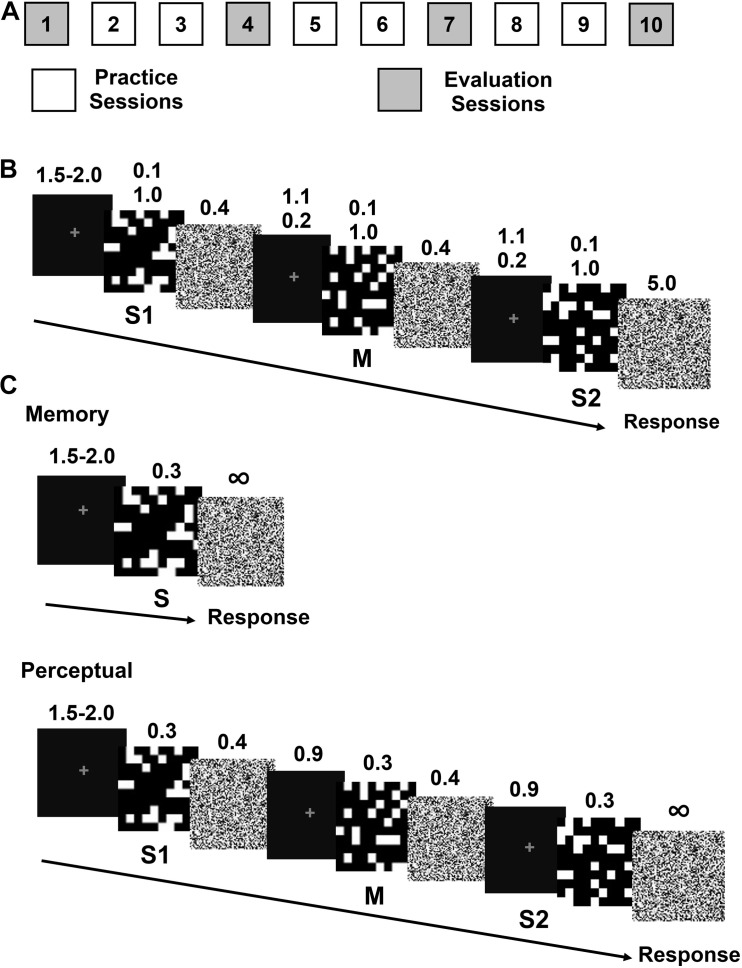
Experimental protocol and trial sequence for the practice and evaluation sessions. **(A)** Sequence of the practice and evaluation sessions. **(B)** An example of a practice trial consisting of a sequence of images: stimulus 1 (S1), perceptual mask (M), and stimulus 2 (S2), each followed by a noise image. The durations of the different trial steps (fixation, f; stimulus, s; noise, n; inter stimulus, i and mask, m) in seconds are specified in the main text. **(C)** An example of a trial of the recognition memory (left) and perception (right) evaluation sessions.

We used black and white scrambled checkerboard-like patterns (10 × 10 squares, 60 exemplars, Figure 1) with 50% of black squares (Montefusco-Siegmund et al., 2018). Participants had no prior experience with either stimuli as specified in the recruitment interview. Stimuli (1.5 × 1.5 visual degrees) were presented on a black background at the center of a 23 inch monitor (ASUS Designo MX239H Monitor, refresh rate 60 Hz) or a LCD 20.1 inch monitor (Dell E207WFPc, refresh rate 60 Hz), at a distance of 57 cm with either MATLAB 2015 (MathWorks, Natick, MA, United States) or NI LabWindows CVI (Austin, TX, United States).

### Procedure

The experiments consisted of two types of sessions: practice and evaluation. The practice sessions were aimed at measuring the impact of encoding time on ODL to provide insight into the source of information required for ODL. By manipulating stimulus duration, our aim was to selectively restrict the available time for visual information extractions based on the assumption about visual processing from prior evidence for faces and object of expertise (Curby and Gauthier, 2009; Dobs et al., 2019). The evaluation sessions were aimed at measuring the effect of encoding time and discrimination practice on perceptual and recognition memory processes, to provide insight into the relationship between information extraction and the perceptual and recognition memory processes during ODL.

In the practice sessions, participants learned to discriminate pairs of unfamiliar scrambled checker-like stimuli. In the evaluation sessions, participants executed either an Old/New memory task or a same/different perceptual task using the same stimuli category from the practice sessions. Participants were randomly assigned to one of four groups: perceptual task-brief stimulus, perceptual task-long stimulus, memory task-brief stimulus and memory task-long stimulus. All the participants completed nine or ten daily sessions, six same/different practice sessions (2, 3, 5, 6, 8, and 9, Figure 1A). The perception group completed four evaluation sessions (1, 4, 7, and 10, Figure 1A) and the memory group completed three evaluation sessions (4, 7, and 10) skipping session 1 because they had no previous experience with the stimuli on the first session. In the following sections we describe the procedure for the practice and evaluation sessions.

#### Practice Sessions

Each trial began with a fixation dot of variable duration (1.5–2.0 s), followed by a sequence of two stimuli S1 and S2, lasting either 0.1 s for brief or 1 s for long stimuli (Figure 1B). To reduce the effect of priming on the second stimulus, a perceptual mask (M), consisting of an exemplar different from S1 and S2, was presented between S1 and S2 for 0.1 s or 1 s for brief and long stimuli, respectively. Stimuli and masks were followed by a noise image (0.4 s for S1 and M and 5 s for S2. The noise masks were built as a high frequency scrambled image from each stimuli. To facilitate the eye fixation on the screen, a fixation dot was shown during the inter-stimulus interval (1.1 s for brief or 0.2 s for long stimuli) between the noise and the succeeding M and S2. The time between the onset of S1 and M and between M and S2 was 1.6 s for all participants (Montefusco-Siegmund et al., 2018).

Seated in a dimly light room, participants began each trial by pressing the center button of a seven button RB-740 Response Pad (Cedrus Corporation, San Pedro, CA, United States). After the second stimuli (S2), they had to respond if the stimuli pair (S1–S2) was perceived as “*same*” or “*different*” by pressing either the left or right buttons as fast and as accurate as possible. Half of the participants responded “*same*” with the right dominant hand and half with the left hand. To avoid the discrimination based on the retinal matching of S1 and S2 on same pairs and to promote object discrimination, S2 was rotated clockwise or counter-clockwise by 90° in a pseudo-random manner. Participants were informed that the third stimulus was rotated.

We built six stimuli lists out of 30 checkerboard-like exemplars, consisting of a random sequence of same and different pairs, with an equal frequency of each exemplar as S1, S2 and M and a 50/50 ratio of same and different pairs. The order of the lists was randomly selected for each participant. Each session of 360 trials, divided into 4 blocks of 90 trials each, lasted approximately 1 h. Between blocks, participants were free to rest and received food and/or beverages upon request.

Participants did not receive feedback on their performance. Because we anticipated a high variability in attention and motivation, all participants were encouraged to perform well at the beginning of each session. Besides, the sensitivity was evaluated at the end of each session and if there was no increase in sensitivity in two successive sessions, participants were told that they should make an effort to be more attentive and perform better in the following session. No quantitative information regarding correct or incorrect responses was provided. Out of 43 participants, five were eliminated because there was no improvement of sensitivity. In consequence, the analysis was done with the remaining 38 participants, 19 with brief and 19 with long stimuli.

#### Evaluation Sessions

To evaluate whether encoding time and discrimination practice shape perception and recognition memory processes during ODL, we obtained the parameters for each process from the ROC curves for the same/different task (Aly and Yonelinas, 2012) and for the Old/New task (Macmillan and Creelman, 2005), respectively.

##### Perception ROC curves

Twenty out of the 38 participants performed a same/different task, 10 with brief stimuli and ten with long stimuli. The stimuli were the same set of 30 checkerboards from the practice sessions. We assembled 4 stimuli lists of 120 trials each, divided in three blocks of 40 trials. For each participant, the order of the lists was randomly selected. To compare the ROC curves from participants that performed the practice sessions with brief and long stimuli, all participants had a S1 and S2 and M duration of 0.3 s. Stimuli and masks were followed by a noise image (0.4 s) for S1 and M and 5 s for S2 and the time between S1 and M; and M and S2 was 1.6 s (Montefusco-Siegmund et al., 2018).

Participants responded after the onset of S2 by selecting one of six options presented at the bottom of the screen (same very sure, same sure, same unsure, different unsure, different sure, and different very sure). Half of the participants had the “*same*” option on the right and the remaining half had them on the left side of the screen. The hand options were counterbalanced between participants.

##### Recognition memory ROC curves

Eighteen out of the 38 participants completed the memory task, nine with brief and nine with long stimuli. ROC memory curves were obtained in the evaluation sessions with a stimulus duration of 0.3 s for all participants. The stimuli were 30 checkerboards from the practice sessions, from here on referred to as “old” and 30 additional checkerboards not included in the practice sessions, from here on referred to as “new.” We built 3 stimuli lists of 120 trials, each with a random sequence of 15 old and 15 new exemplars, divided in three blocks of 40 trials. For each participant, the order of the lists was randomly selected. Each trial began with a fixation dot of a variable duration (1.0–1.5 s), followed by the stimuli (S, 0.3 s) and a noise mask (Figure 1C). Participants responded after the onset of S according to six options presented at the bottom of the screen (old very sure, old sure, old unsure, new unsure, new sure, and new very sure). Half of the participants had the “old” option on the right and the remaining half on the left side of the screen. Participants were instructed to respond if each stimulus was presented during the practice sessions (old) or not (new). The hand options were counterbalanced between participants.

#### Sensitivity

As previously described (Montefusco-Siegmund et al., 2018), the sensitivity was quantified using the signal detection theory. We obtained the discrimination index (*d* prime, *d*′) or sensitivity (Green and Swets, 1966) assuming the differencing strategy (Macmillan and Creelman, 2005), using the Palamedes toolbox (AL_SDT_1AFCsameDiff_DiffMod_PHFtoDP routine)^1^ (Kingdom and Prins, 2010), written in MATLAB (MathWorks Inc., Natick, MA, United States). The mean sensitivity values were fitted to a quadratic equation, the fitted equations are described in the Supplementary Material.

#### ROC Parameters for Recognition Memory and Perception

For the memory task, we obtained the individual ROC plots by accumulating the responses from the more confident “new” responses to the more confident “old” responses to new stimuli (pNew/New), from here on defined as the target, and from the more confident “New” responses to the more confident “Old” responses to old stimuli (pNew/Old), from here on defined as the lure. The individual plots for the targets versus lures where fitted to the dual-process signal detection model with maximum likelihood estimation using the MATLAB ROC toolbox (Koen et al., 2017). From the ROC curve fitting we obtained the parameters of the state process of *detection of new* (pNew) corresponding to the *y*-axis crossing and *recall* (pOld), corresponding to the superior *x*-axis crossing and the strength process of *familiarity* (Fam), corresponding to the curvature of the ROC plot.

For the perceptual task, we obtained the individual ROC plots by accumulating the responses from the more confident “different” responses to the more confident “same” responses to different stimuli pairs (pDiff/Diff), designated as the target, and from the more confident “same” responses to the more confident “different” responses to equal stimuli pairs (pSame/Diff), designated as the lure. From the ROC curve fitting we obtained the parameters of the state process of *perceive different* (pDiff) corresponding to the *y*-axis crossing and the *perceive same* (pSame), corresponding to the superior *x*-axis crossing and the strength process of *know* (Know), corresponding to the curvature of the target versus lure plot.

#### Statistical Analysis

Statistical differences were estimated with Bayesian analysis using the Bayes Factor Toolbox for MATLAB [https://github.com/klabhub/bayesFactor written by Bart Krekelberg based on Rouder et al. (2012)]. Differences in performance were evaluated by a one or two factor ANOVA. The statistical differences of the ROC parameters between sessions were evaluated by a two factor ANOVA (stimulus duration × evaluation session) for the perception and the memory groups. Main effects for the ANOVA were estimated as the ratio of the Bayes Factors for the full model and the restricted model calculated excluding the main effect factor. A Bayes Factor for the alternative hypothesis (H1) greater than 10 is considered a strong evidence to support H1, where H1 is greater than ten times more likely than H0 or in other terms, the probability for H1 from the data is 90%. A Bayes Factor between 2 and 10 is considered a moderate evidence to support H1, where H1 is between twice and ten times more likely than H0 or in other terms, the probability for H1 from the data is <90% (van Doorn et al., 2020). We established a BF greater than ten as strong evidence for the alternative hypothesis and considered that BF between 2 and 10 as moderate, non-conclusive evidence for the effect.

Differences between means were assessed as the Bayes factor for paired or unpaired *t*-tests. To evaluate the association between the perception and memory parameters with performance and extent of practice, we computed the Bayes factor for the Pearson product-moment correlation coefficient. A strong correlation was defined when *r*^2^ values were equal or greater than 0.5 and a moderate correlation when *r*^2^ values were between 0.45 and 0.5. Unless otherwise specified, all values are reported as mean + SD of the mean in the main text. All figures values are reported as mean + SEM. Statistical significance for *t*-tests was set to a probability from data ≥0.90 (BF ≥ 10) and for correlations was set to probability for the alternative hypothesis (pH1) ≥ 0.90 (BF ≥ 10). Again, a BF for the alternative hypothesis between 2 and 10 was considered as moderate non-conclusive evidence.

## Results

The aims of this work were to evaluate if the early and late visual processes are required for ODL and whether encoding time and discrimination practice shape perception and recognition memory processes during ODL.

### Visual Processing Time and ODL

To evaluate if early and late visual processing are sufficient for ODL, we measured the performance as the participant’s sensitivity at each practice session. We first verified if the type of evaluation had an effect on performance in the practice sessions. A one factor (evaluation type; perception, memory) ANOVA confirmed no difference in the performance in the practice sessions due to the evaluation type (*F*(1, 226) = 1.26, BF_10_ = 0.16 for a difference in dprime between evaluation types). Thus, performance from the practice sessions was pooled in two groups, brief and long stimuli, for further analysis of the ODL.

**FIGURE 2:**
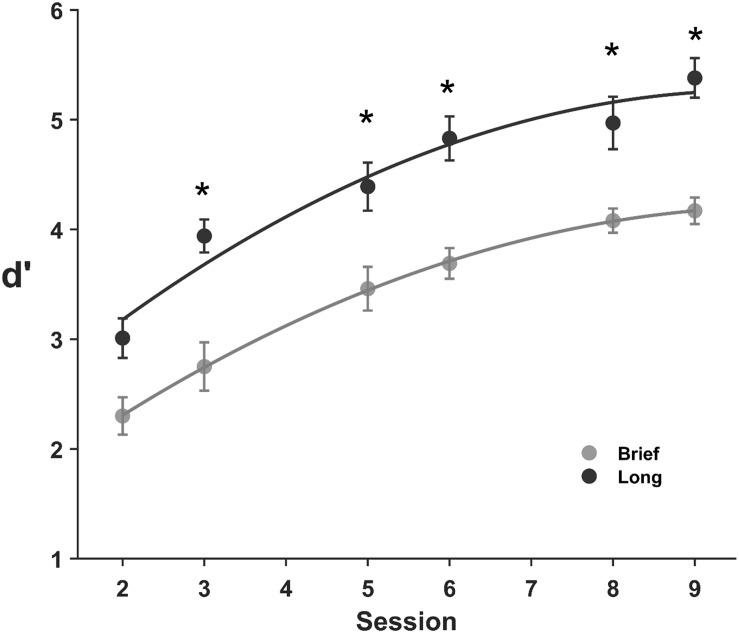
Discrimination learning of complex images for brief and long stimuli. Mean *d*′ values across practice sessions for brief (0.1 s, gray) and long (1 s, black) stimuli. Error bars are SE of the mean. Asterisks indicate statistical differences between means (*p ≤ 0.005).

The performance across practice sessions with brief and long stimuli (Figure 2) exhibits a progressive improvement across sessions, although it was lower for brief stimuli in the majority of sessions and showed a similar overall improvement with brief and long stimuli across sessions. Specifically, the mean sensitivity for brief stimuli increased by 86% from *M* = 2.30, SD = 0.76 in the first session to *M* = 4.17, SD = 0.54 in the ninth session. Similarly, the sensitivity for long stimuli increased by 78% from *M* = 3.02, SD = 0.77 in the first session to *M* = 5.38, SD = 0.77 in the ninth session. The 2 (encoding time; brief, long) × 6 (practice session; 2, 3, 5, 6, 8, 9) ANOVA on the sensitivity showed main effects of both encoding time (*F*(1, 224) = 11.0, BF_10_ = 2.9 × 10^15^) and practice session (*F*(1, 224) = 99.2, BF_10_ = 8.7 × 10^26^), but no interaction between encoding time and practice session (*F*(1, 224) = 0.54, BF_10_ = 0.09). A brief encoding time reduced performance compared to a long encoding time in sessions 3, 5, 6, 8, and 9 (BF_10_ = 288, 10.6, 510, 21.2, 6380 for a difference in dprime between brief and long stimuli for sessions 2, 3, 5, 6, 8, and 9, probability from data = 0.87, 0.99, 0.91, 0.99, 0.95, and 0.99, respectively), and there was a moderate effect on session two (BF_10_ = 6.6, probability from data = 0.87, unpaired *t*-test).

As expected, discrimination practice increased performance from the second practice session (sessions 3, 5, 6, 8, 9, BF_10_ > 1.0 × 10^4^ for a difference in dprime between the first and subsequent practice sessions, probability from data > 0.99, paired *t*-test). Interestingly, our data does not support a difference in the overall increase of performance across practice sessions (*M* = 1.86, SD = 0.62 and, *M* = 2.37, SD = 0.74 for brief and long stimuli, respectively) with a moderate BF_10_ = 2.3 for a difference in the total performance improvement across sessions for brief and long stimuli, probability from data = 0.70, unpaired *t*-test. Taken together, these results demonstrate that brief stimuli which constrains feature extraction, reduces the sensitivity and that discrimination practice improves performance, and more interestingly, that the overall ODL was not dependent on the encoding time over six practice sessions.

### Perception and Recognition Memory Modulation During ODL

To evaluate whether encoding time and discrimination practice modulate the perception and recognition memory processes, we obtained the ROC parameters for these processes during visual ODL and expertise acquisition. For recognition memory and perception, the gradual “strength” processes (*familiarity* and *know*, respectively) and the all or none “state” processes (*recall* and *perceive*, respectively) have been typically estimated with meaningful familiar stimuli (Broder and Schutz, 2009; Aly and Yonelinas, 2012). Here, by manipulating the stimulus duration and the extent of practice, we investigated whether encoding time and discrimination practice modulate the perception and recognition memory processes during ODL. These processes were evaluated every two practice sessions: two groups on perception (same/different task: brief and long stimuli) and two groups on memory (Old/New task: brief and long stimuli) with an equal stimulus duration for all participants. We hypothesize that if perceptual processes are the main factors modulated during discrimination learning, then practice will promote *know* and *perceive* with brief and long stimuli, respectively, and these processes will associate with performance and ODL. On the contrary, if recognition memory processes are the main factors modulated during discrimination learning, then practice with brief stimuli will promote *familiarity* and practice with long stimuli will promote *recall* and these processes will associate with performance and ODL.

#### Perceive and Know Modulation During ODL

**FIGURE 3:**
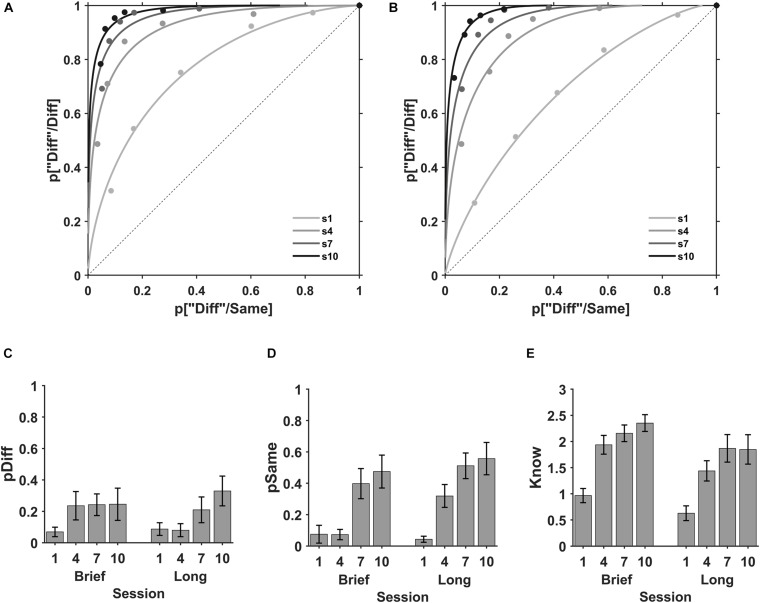
Perceive and Know during discrimination learning. ROC plots for the mean of the individuals during the perception evaluation sessions. Sessions first (s1, light gray), fourth (s4, gray), seventh (s7, dark gray), and tenth (s10, black) for **(A)** brief and **(B)** long exposure durations. The mean *perceive different* (pDiff), *perceive same* (pSame), and *know* parameters during the perception evaluation sessions. **(C)** State process “diff” (pDiff) in evaluation sessions first (1), fourth (4), seventh (7), and tenth (10) with brief (left) and long (right) stimulus durations l. **(D)** State process “same” (pSame) in the evaluation sessions with brief (left) and long (right) stimulus durations. **(E)** Strength *know* process in evaluation sessions with brief (left) and long (right) stimulus durations. Error bars are SE of the mean.

In the perceptual evaluation sessions, 20 participants from the brief (*N* = 10) and long (*N* = 10) stimulus duration groups, performed a same-different task with a stimulus duration of 0.3 s (Figure 1C). ROC plots for the mean of individual data at each evaluation session are shown in Figure 3. The *know* process, representing the plot curvature, was small in the first session and increased progressively as the practice increased for brief and long stimuli. *Perceive same* (pSame, superior *x*-axis crossing) and *perceive different* (pDiff, *y*-axis crossing) processes show variable modulation as practice increased for both stimuli durations.

The perception parameters *perceive different*, *perceive same* and *know*, obtained by fitting the individual ROC plots for each participant at each evaluation session are shown in Figure 3. P*erceive different* was small in the first session and exhibited a small increase with practice for brief and long stimuli (Figure 3C). For brief stimuli the values for the first, fourth, seventh, and tenth evaluations sessions were *M* = 0.069, SD = 0.096; *M* = 0.235, SD = 0.286; *M* = 0.242, SD = 0.218; and *M* = 0.245, SD = 0.324, respectively (Figure 3C, left) and for long stimuli *M* = 0.087, SD = 0.127; *M* = 0.080, SD = 0.131; *M* = 0.209, SD = 0.259; and *M* = 0.330, SD = 0.300, respectively (Figure 3C, right). The 2 (encoding time; brief, long) × 4 (evaluation session; 1, 4, 7, 10) ANOVA for the *perceive different* showed an effect of session number (*F*(1, 76) = 7.23, BF_10_ = 12.7), no effect of stimulus duration (*F*(1, 76) = 0.69, BF_10_ = 0.18) and no interaction between session and encoding time (*F*(1, 76) = 0.51, BF_10_ = 0.22). For *perceive different*, the paired comparisons show that our data does not support a significant modulation of *perceive different* with practice between the first and the fourth session (BF_10_ = 0.23, probability from data = 0.19), but there is a moderate support for the modulation of practice in sessions 7th and 10th (BF_10_ = 3.54 and 6.6, and a probability from data = 0.78 and 0.87, *t*-test).

Conversely, *perceive same* was small in the first session and exhibited a variable increase with practice for brief and long stimuli (Figure 3D). *Perceive same* for the first, fourth, seventh, and tenth evaluations sessions were *M* = 0.075, SD = 0.180; *M* = 0.073, SD = 0.104; *M* = 0.398, SD = 0.304; and *M* = 0.475, SD = 0.331, respectively (Figure 3D, left) for brief and *M* = 0.043, SD = 0.062; *M* = 0.319, SD = 0.231; *M* = 0.511, SD = 0.258; and *M* = 0.557, SD = 0.326, respectively (Figure 3D, right) for long stimuli. The ANOVA showed a main effect of session (*F*(1, 76) = 26.16, BF_10_ = 4.66 × 10^6^), no effect of encoding time (*F*(1, 76) = 0.38, BF_10_ = 0.76) and no interaction of session and encoding time (*F*(1, 76) = 0.19, BF_10_ = 0.15). Paired comparisons show that practice increases *perceive same* after four practice sessions (4, 7, and 10, BF_10_ = 2.0, 1.73 × 10^3^ and 2.01 × 10^3^ respectively, probability from data = 0.67, 0.99, 0.99, respectively, paired *t*-test).

Likewise, the *know* parameter increased with practice for brief and long stimuli (Figure 3E). The *know* parameter for sessions first, fourth, seventh and tenth were *M* = 0.967, SD = 0.429; *M* = 1.936, SD = 0.567; *M* = 2.157, SD = 0.501; and *M* = 2.351, SD = 0.506, respectively (Figure 3E, left) with brief stimuli and *M* = 0.629, SD = 0.445; *M* = 1.438, SD = 0.613; *M* = 1.868, SD = 0.834; and *M* = 1.848, SD = 0.889, respectively (Figure 3E, right) for long stimuli. The ANOVA showed a main effect of session (*F*(1, 76) = 21.47, BF_10_ = 4.45 × 10^6^), a moderate effect of encoding time (*F*(1, 76) = 1.75, BF_10_ = 6.35) and no interaction between session and encoding time (*F*(1, 76) = 0.05, BF_10_ = 0.14). Thus, practice increased the *know* parameter after two practice sessions (4, 7, 10, BF_10_ = 3.94 × 10^3^, 5.77 × 10^5^, and 9.52 × 10^5^, respectively, probability from data ≥ 0.99). Moreover, our data does not support a difference in the *know* parameter between brief and long stimuli in sessions 1, 4, 7, and 10 (BF_10_ = 0.63, 0.53, 1.01, 0.72, probability from data = 0.39, 0.35, 0.52, 42, respectively.

In summary, these results show that discrimination practice increased the state *perceive same* and strength *know* parameters after four and two practice sessions, respectively. Besides, *perceive different* shows a non-conclusive modulation by practice after four practice sessions. In contrast, the perception parameters were not modulated by the encoding time in the discrimination practice.

#### Recall and Familiarity Modulation During ODL

**FIGURE 4:**
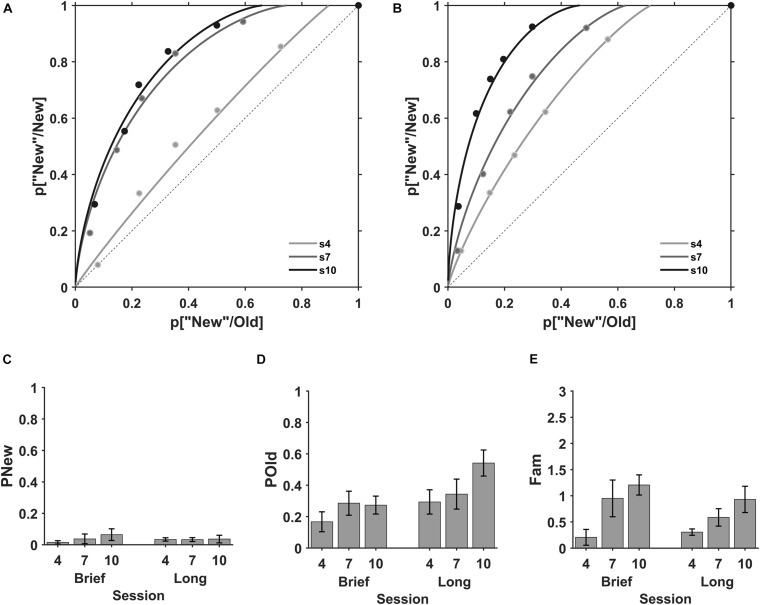
Recall and Familiarity during discrimination learning. ROC plots for the mean of the individuals in the memory evaluation sessions. Sessions fourth (s4, gray), seventh (s7, dark gray) and tenth (s10, black) for **(A)** brief and **(B)** long stimulus durations. Mean *detection of new* (pNew), *recall* (pOld) and *familiarity* (Fam). **(C)** The state process pNew in sessions four (4), seven (7) and ten (10) for brief (left) and long (right) stimulus durations. **(D)** The state process pOld across evaluation sessions for brief (left) and long (right) stimulus durations. **(E)** The strength process Fam across evaluation sessions for brief (left) and long (right) stimulus durations. Error bars are SE of the mean.

The explicit memory for the viewed stimuli can be evaluated using the recognition memory test. Here, we evaluated the *recall* and *familiarity* processes in three evaluation sessions, interspersed every two practice sessions, where eighteen participants from the brief (*N* = 9) and long (*N* = 9) stimulus duration groups performed an Old/New task with previously viewed exemplars from the practice sessions (old) and new exemplars (new, Figure 1C) using the same stimulus duration of 0.3 s. The ROC plots for the mean of individual data at each evaluation session suggest an increase of the *familiarity* (plot curvature) with discrimination practice (Figure 4).

The *detection of new* (pNew), *recall* (pOld), and *familiarity* (Fam) were obtained from the fitting of the individual ROC plots at each evaluation session (Figure 4). The *detection of new* in sessions four, seven and ten were *M* = 0.015, SD = 0.032, *M* = 0.037, SD = 0.093, and *M* = 0.045, SD = 0.102, respectively (Figure 4C, left) with brief stimuli and *M* = 0.034, SD = 0.032, *M* = 0.033, SD = 0.036, and *M* = 0.036, SD = 0.073, respectively (Figure 4C, right) with long stimuli.

The ANOVA showed no effect of session (*F*(1, 50) = 0.006, BF_10_ = 0.26), encoding time (*F*(1, 50) = 0.41, BF_10_ = 0.21) and no interaction between session and encoding time (*F*(1, 50) = 0.41, BF_10_ = 0.26). Thus, discrimination practice and encoding time did not modulate the *detection of new*.

In contrast, the recall (pOld) in sessions four, seven and ten were similar (*M* = 0.164, SD = 0.192; *M* = 0.286, SD = 0.230; *M* = 0.239, SD = 0.193, respectively; Figure 4D, left) for brief stimuli and exhibited a small increase in sessions four, seven and ten (*M* = 0.234, SD = 0.232; *M* = 0.344, SD = 0.287; *M* = 0.542, SD = 0.249, respectively; Figure 4D, right) for long stimuli. The ANOVA showed no effect of session (*F*(1, 50) = 5.58, BF_10_ = 1.6), a moderate effect of encoding time (*F*(1, 50) = 0.04, BF_10_ = 4.6) and no interaction between session and encoding time (*F*(1, 50) = 1.36, BF_10_ = 0.37). Unpaired comparisons between brief and long stimuli for each session show that our data does not support a modulation of *recall* by the encoding time in sessions 4th and 7th (BF_10_ = 0.41, 0.55, probability from data = 0.29, 0.35, respectively), but there is a moderate support for the modulation of encoding time in session 10th (BF_10_ = 5.0, probability from data = 0.83).

Finally, the *familiarity* showed a progressive increase with session number for brief and long stimuli. The *familiarity* in sessions four, seven and ten was *M* = 0.156, SD = 0.451, *M* = 0.953, SD = 1.053, *M* = 1.111, SD = 0.610, respectively for brief stimuli (Figure 4E, left) and *M* = 0.307, SD = 0.183, *M* = 0.588, SD = 0.498, *M* = 0.932, SD = 0.752, respectively for long stimuli (Figure 4E, right). The ANOVA showed a main effect of session (*F*(1, 50) = 4.55, BF_10_ = 76.1), no effect of encoding time (*F*(1, 50) = 0.25, BF_10_ = 0.25) and no interaction between session and encoding time (*F*(1, 50) = 0.64, BF_10_ = 0.25). Hence, these results show that there is a strong support for the modulation of *familiarity* after six practice sessions (session 10th, F_10_ = 297.0 and a probability from data ≥ 0.99) and a moderate support for the modulation after four practice sessions (session 7th, BF_10_ = 3.99 and a probability from data = 0.80).

In summary, these results show that discrimination practice increased the strength *familiarity* parameter after four practice sessions. Besides, the state *recall* parameter exhibited a non-conclusive modulation by the encoding time in the discrimination practice.

### Association Between the Perception and Recognition Memory Processes With Discrimination Practice and Learning

In the previous section, we showed that discrimination practice is the main factor that shapes the perceptual and recognition memory processes. To better understand the relationship between practice/encoding time and perception/recognition memory processes, we evaluated the association between them by performing a Pearson correlation. We used the values of the immediately preceding practice session, except for the first perceptual evaluation session which was done with the sensitivity of the immediately succeeding practice session (session 2).

#### Perception

**FIGURE 5:**
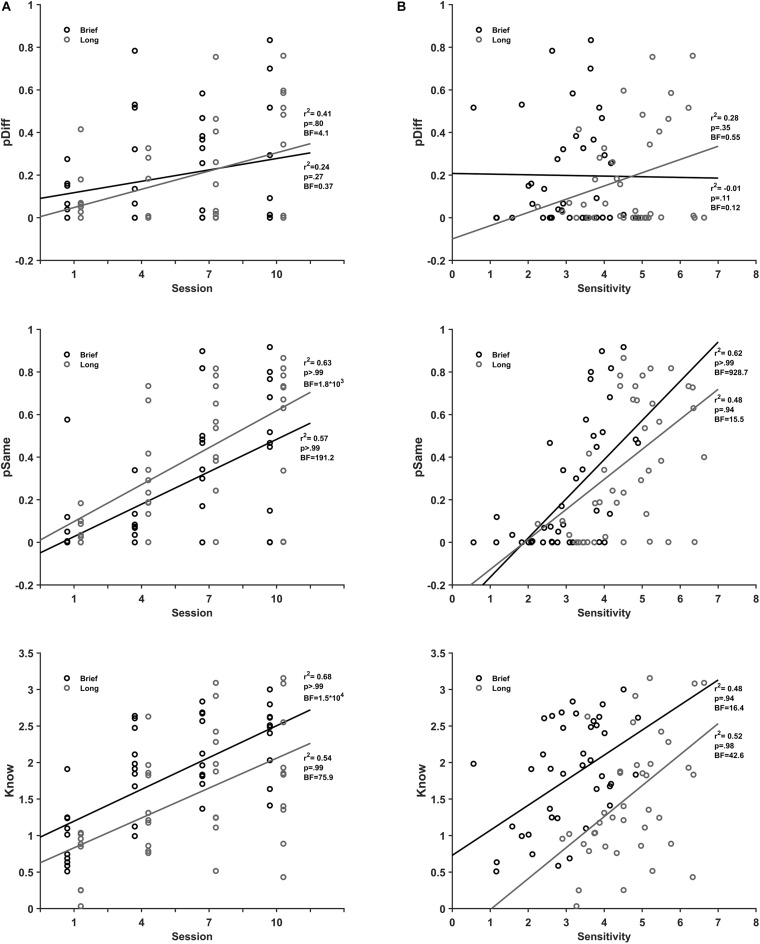
Perceive and Know association with discrimination practice and sensitivity. **(A)**. Mean pDiff (black), pSame (dark gray), and Know (gray) parameters for brief (top) and long (bottom) stimulus durations as a function of session number. **(B)** pDiff (black), pSame (dark gray), and Know (gray) parameters for brief (top) and long (bottom) stimulus durations as a function of sensitivity.

The scatterplots of the perception parameters as a function of practice sessions (Figure 5A) show that *perceive same* and *know* parameters correlate positively with the extent of practice. There was a strong correlation of the *perceive same* (*r*^2^(38) = 0.57, BF_10_ = 191.2, pH1 > 0.99) and *know* (*r*^2^(38) = 0.68, BF_10_ 1.5 × 10^4^, pH1 ≥ 0.99), and no correlation of the *perceive different* (*r*^2^(38) = 0.24, BF_10_ = 0.37, pH1 = 0.27) with practice for brief stimuli. Similarly, for long stimuli there was a strong correlation of the *perceive same* (*r*^2^ (38) = 0.63, BF_10_ = 1.8 × 10^3^, pH1 > 0.99) and *know* (*r*^2^(38) = 0.54, BF_10_ = 75.9, pH1 = 0.99) and no correlation of the *perceive different* with moderate support from data (*r*^2^(38) = 0.41 BF_10_ = 4.1 and pH1 = 0.80) with practice.

Likewise, the scatterplots of the perception parameters as a function of sensitivity (Figure 5B) show that *perceive same* and *know* parameters correlate positively with performance in the practice sessions. There was a strong correlation of *perceive same* (*r*^2^(38) = 0.62, BF_10_ = 928.7, pH1 ≥ 0.99), and *know* (*r*^2^(38) = 0.48, BF_10_ = 16.4, pH1 = 0.94), and no correlation of *perceive different* (*r*^2^(38) = −0.01, BF_10_ = 0.12, pH1 = 0.11) with sensitivity for brief stimuli. For long stimuli, there was a moderate correlation with support from data for *perceive same* (*r*^2^(38) = 0.48, BF_10_ = 15.5, pH1 = 0.94), a strong correlation of *know* (*r*^2^(38) = 0.52, BF_10_ = 42.6, pH1 = 0.98), and no correlation of *perceive different* (*r*^2^(38) = 0.28, BF_10_ = 0.55, pH1 = 0.35) with sensitivity. In summary, *perceive same* and *know* processes are associated with both discrimination practice and sensitivity from the practice sessions for brief and long encoding times. In contrast, *perceive different* is not associated with discrimination practice and encoding time.

#### Recognition Memory

**FIGURE 6:**
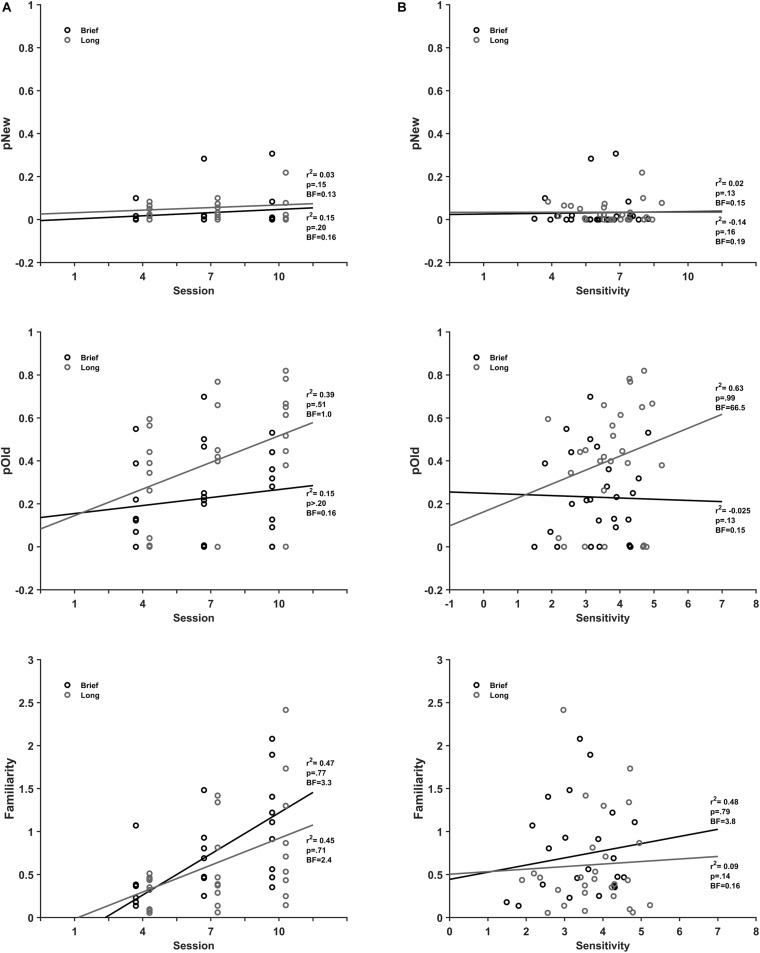
Recollection and Familiarity association with discrimination practice and sensitivity. **(A)** Mean pOld (dark gray), pNew (black), and Fam (gray) parameters for brief (top) and long (bottom) stimulus durations as a function of discrimination session. **(B)** Mean pOld dark gray), pNew (black) and Fam (gray) parameters for brief (top) and long (bottom) stimulus as a function of discrimination sensitivity.

The scatterplots of the recognition memory parameters from the evaluation sessions as a function of the practice session (Figure 6A) show that none of the recognition memory parameters correlate with the extent of practice. There was no correlation of *detection of new* (*r*^2^(25) = 0.15, BF_10_ = 0.20, pH1 = 0.16), *recall* (*r*^2^(25) = 0.15, BF_10_ = 0.20, pH1 = 0.16) and a moderate correlation with moderate support from data for *familiarity* (*r*^2^(25) = 0.47, BF_10_ = 3.3, pH1 = 0.77) with practice for brief stimuli. Likewise, for long stimuli there was no correlation of the *detection of new* (*r*^2^(25) = 0.03, BF_10_ = 0.15, pH1 = 0.13), *recall* (*r*^2^(25) = 0.39, BF_10_ = 1.0, pH1 = 0.51) and a moderate correlation with moderate support from data for *familiarity* (*r*^2^(25) = 0.45, BF_10_ = 2.4, pH1 = 0.71).

The *recall* process exhibited different correlations with sensitivity for brief and long stimuli (Figure 6B). There was no correlation of the *detection of new* (*r*^2^(25) = 0.02, BF_10_ = 0.15, pH1 = 0.13), *recall* (*r*^2^(25) = −0.025, BF_10_ = 0.15, pH1 = 0.13) and no correlation of *familiarity* (*r*^2^(25) = 0.09, BF_10_ = 0.16, pH1 = 0.14) with sensitivity for brief stimuli. In contrast, there was a strong correlation of *recall* (*r*^2^(25) = 0.63, BF_10_ = 66.5, pH1 = 0.99) and no correlation of the *detection of new* (*r*^2^(25) = −0.14, BF_10_ = 0.19, pH1 = 0.16) and moderate correlation with moderate statistical support for *familiarity* (*r*^2^ (25) = 0.48, BF_10_ = 3.8, pH1 = 0.79) with sensitivity for long stimuli.

These results show that *familiarity* is moderately associated with discrimination practice for brief and long stimuli, although with moderate statistical evidence from data. Moreover, the *recall* as well as the *familiarity* processes associate with performance in the discrimination sessions, although the later with a moderate statistical evidence. In contrast, our results show that discrimination practice and sensitivity do not associate to the *detection of new* process.

## Discussion

This study’s aims were to evaluate whether early and late visual perceptual processes are required for ODL and perceptual expertise acquisition as well as to assess the extent of modulation of perceptual and recognition memory processes during discrimination practice and ODL by encoding time and the amount of practice. First, we find an inferior performance with brief stimulus duration, regardless of practice, suggesting that fine information extraction during the late stages of visual processing is required for accurate discrimination. Interestingly, we find that the overall ODL, defined as the difference in performance across sessions, was equivalent for brief and long stimulus duration, suggesting that early perceptual processing stages, likely including image decoding and coarse information extraction, are sufficient for discrimination learning across the six practice sessions.

Secondly, we find that discrimination practice enhances *perceive same* and *know* processes of perception, and both processes associate with discrimination practice and performance. For recognition memory, discrimination practice enhances *familiarity* and the latter is moderately associated with practice and performance. In addition, the long encoding time enhances *recall* and the latter is moderately associated with discrimination performance for long encoding times. Taken together, our results suggest a distinct modulation of perception and recognition memory processes by discrimination practice and encoding time, indicating the engagement of both processes during discrimination practice but a dissimilar relationship between these processes and performance in the practice sessions. Because the state and strength perceptual processes exhibited a strong association with discrimination practice and performance during ODL, our results suggest that practice improves performance mostly through the modulation of the perceptual processes. In the following paragraphs we relate these findings to prior results on discrimination learning and expertise and discuss their significance and limitations.

The inferior discrimination performance with brief stimuli durations is consistent with previous studies in detection, individuation and discrimination tasks. For example, performance with brief stimuli was lower in non-face object categorization (Tanaka, 2001), car discrimination (Curby and Gauthier, 2009) and object or face identification (Grill-Spector and Kanwisher, 2005). Specifically, our results are consistent with those of Curby and Gauthier (2009; Figure 4) where car experts show a sustained greater discrimination sensitivity than novices at encoding times from 50 to 1,000 ms.

Although no studies have evaluated the visual processing of unknown complex stimuli, previous work with faces (Goffaux et al., 2011; Mohsenzadeh et al., 2018; Dobs et al., 2019) and scenes (Musel et al., 2014; Loschky et al., 2015) suggests that the processing in the present study likely implicates image decoding plus coarse and fine information extraction. For faces, the quintessential object of expertise for humans, image decoding occurs before 100 ms, coarse feature extraction around 100 ms and fine feature extraction such as identity information occurs after 100 ms (Goffaux et al., 2011; Dobs et al., 2019). Since the timing for image decoding did not differ between familiar and unfamiliar objects (Dobs et al., 2019), we assumed an equal image decoding time across all practice sessions. Moreover, because coarse and fine information extraction for unfamiliar stimuli occurs at a later time from stimuli onset compared to familiar stimuli (Curby and Gauthier, 2009, 2010; Dobs et al., 2019), the lower performance with brief stimuli may partly arise from an incomplete fine information extraction. In other words, the superior performance on the majority of sessions with long encoding time can be attributed to a greater time for information extraction, directly impacting in the correct responses.

The unsurprising improved discrimination performance with increasing amounts of practice corroborates the ubiquitous effects of practice in performance and expertise acquisition (Shen et al., 2014). For example, greater accuracy was observed after training with an initially unfamiliar stimuli in discrimination (de Beeck et al., 2006; Montefusco-Siegmund et al., 2018), individuation (Bukach et al., 2012), object recognition (Gauthier and Tarr, 1997; Gauthier et al., 1998), and subordinate categorization (Tanaka et al., 2005). Our results show that sensitivity follows a similar progression throughout the practice sessions with a greater increase in the initial sessions and the onset of asymptotic performance in the last sessions, particularly with brief stimuli. Since the asymptotic performance was not achieved, further studies are needed to evaluate this aspect of the learning process.

Nevertheless, two mechanisms likely account for the progression of performance with discrimination practice; a modification of the stimuli representations and an earlier availability of the coarse and fine information from stimulus onset. As for the representations, previous work showed that discrimination practice with initially unfamiliar stimuli modifies the spatial distribution of object selective areas (de Beeck et al., 2006) and increases the resolution of the perceptual representations for those features that are relevant to the perceptual (de Beeck et al., 2006; Raiguel et al., 2006) and categorization tasks (Folstein et al., 2013, 2015). A greater resolution of the representations for the whole stimuli category also is supported by a nearly full generalization of discrimination learning to novel exemplars of the category (Gauthier et al., 1998; Tanaka et al., 2005; Baeck et al., 2012; Montefusco-Siegmund et al., 2018).

In addition, the increase in performance is attributed to the availability of stimuli information at earlier stages of visual processing (Curby and Gauthier, 2009; Cichy et al., 2014; Mohsenzadeh et al., 2018; Dobs et al., 2019). In consequence, after a long practice more information can be extracted when encoding times are restricted, which could contribute to increased performance with practice. Other mechanisms that explain the higher performance associated with high levels of exposure and experience are higher resolution of short-term memory (STM) representations (Scolari et al., 2008) and greater encoding and consolidation in short term memory (Jacob et al., 2013; Xie and Zhang, 2017). Further studies are needed to distinguish between effects of discrimination practice on perceptual processing and STM.

An interesting finding of our work is the similar ODL with brief and long perceptual encoding times across the six practice sessions. To the best of our knowledge, no study has tested the effect of encoding times and the underlying stages of visual processing in ODL. Our results show for the first time that a brief encoding time of 100 ms, likely including image decoding and early stages of information extraction (Cichy et al., 2014; Mohsenzadeh et al., 2018; Dobs et al., 2019), is sufficient for ODL of a multiexemplar category of complex stimuli. This result suggests that the information available in the first 100 ms is sufficient to shape the spatial distribution of selective areas, to enhance the resolution of the representations for the stimuli category and to reduce the latency of the coarse and fine information extraction. Moreover, additional studies should evaluate discrimination performance with shorter encoding times to examine the involvement of earlier stages of visual processing on ODL. A limitation of this study is that asymptotic performance is not achieved due to the low number of practice sessions, so we cannot rule out that maximum performance will differ with more practice and leads to higher ODL with long encoding times. Further studies should evaluate performance with longer practice.

On the modulation of the perception processes during discrimination practice and ODL, the *perceive same* and *know* processes are both shaped by discrimination practice, indicating that behavioral decisions in the perceptual task are based on both, “state” process of perception of images that vary in specific details, and a “strength” process of perception of images that vary in overall relational information (Aly and Yonelinas, 2012). Because the task in the practice and perceptual evaluation sessions was the same, except for the duration of the stimuli, we cannot generalize the modulation of perceptual processes to different conditions. Thus, additional studies should examine if the perceptual modulation extends to other perceptual tasks. Besides, the *perceive different* shows inconclusive statistical evidence for the modulation by practice, probably due to a weaker effect of practice on this parameter and a low number of participants.

More interestingly, both *perceive same* and *know* processes increase with greater practice and performance, suggesting that practice shapes the discrimination performance through the modulation of the perception processes. Consequently, the improved performance with practice likely stems from boosted *perceive same* and *know* processes, in agreement with a more efficient perceptual processing (Curby and Gauthier, 2010; Richler et al., 2011; Harel, 2016). Moreover, the modulation of the perception processes is similar for brief and long encoding times, suggesting that image decoding and coarse information extraction are sufficient for the shaping of perceptual parameters, though they are not sufficient for accurate discrimination. We conclude that discrimination practice is a main factor shaping perceptual processes for stimuli durations equal to or greater than 100 ms, suggesting that the early stages of visual processing, such as image decoding and coarse information extraction, are sufficient to shape the representations for the stimuli category and to shift the extraction of coarse and fine features to earlier processing times during ODL. Thus, the practice-dependent modulation of the behavioral responses in a same/different task is based on both the detection of specific details of the stimuli and the sensation of general similarities between the stimuli, represented in the activity of the parietal and occipital cortices, respectively (Aly et al., 2014). Additionally, the faster consolidation (Xie and Zhang, 2017) and greater resolution (Brady et al., 2016) of STM for familiar stimuli might contribute to greater performance.

On the modulation of the recognition memory processes, discrimination practice enhances *familiarity*, a “strength” signal indicating prior occurrence based on an overall awareness of stimuli (Evans et al., 2011) after six practice sessions. However, the statistical evidence for an increase in *familiarity* with greater discrimination practice for brief and long stimuli and with greater performance for long stimuli is moderate, probably due to a weak effect and a low number of participants. These results suggest that discrimination practice improves general awareness of the previous occurrence of stimuli and that this signal probably increases with higher performance for long stimuli.

Moreover, the *recall* is moderately modulated by the encoding time and an improvement in performance is accompanied by a greater *recall* for long encoding time, suggesting the acquisition of a detailed memory for the viewed stimuli with long stimulus exposure. Again, the statistical evidence for the modulation of *recall* by encoding time is moderate. Taken together, these results suggest that the practice of a perceptual discrimination task engages both *familiarity* and *recall* recognition memory processes. Because the statistical support is non-conclusive, further studies with greater number of participants and practice must be carried out to corroborate the modulation of the recognition memory processes during a perceptual discrimination task.

The development of a recognition memory with stimuli exposure is consistent with previous studies showing a great long term memory capacity for a single exposure to 10.000 scenes for 10 s that was sufficient for over 80% recognition even after 3 days (Standing et al., 1970) and a detailed recognition memory for 2.500 images shown for 3 s (Brady et al., 2008). However, the latter were memory studies in which participants had to memorize meaningful stimuli involving semantic representations (Drew et al., 2018) that increase the *familiarity* and *recall* in memory tasks. Moreover, the practice-dependent increase in *familiarity* and *recall* is consistent with a greater recognition memory as a function of total exposure time (Martini and Maljkovic, 2009).

However, our results show a weak acquisition of recognition memory across the perceptual task, in agreement with studies of that compared recognition memory of perceptual experts – such as cytologists, radiologists – and naive participants which showed that experts are slightly better than non-experts at remembering images of the expertise category, but this memory is substantially inferior compared to the one for everyday scenes and objects (Evans et al., 2011), suggesting that perceptual discrimination and identification tasks do not necessarily promote the acquisition of a recognition memory. In contrast, memory studies have shown that experts have superior recognition memory for the expertise category (Nyhus and Curran, 2009; Herzmann and Curran, 2011), but these studies were conducted with stimuli categories that include semantic representations, which enhance deep coding leading to superior *familiarity* and *recall* (Buchsbaum et al., 2015). Thus, the practice of a perceptual task does not necessarily foster the acquisition of a recognition memory unlike what happens in a memory task.

Regarding the effect of encoding time on the *recall* and the association of *recall* to discrimination performance for long encoding times, our results are consistent with the evidence from memory tasks where long study times promote recognition memory, while short times promote implicit memory or priming (Voss and Gonsalves, 2010). Our data show that long encoding times promote *recall* after six practice sessions, probably because it is a perceptual rather than a memory task, as well as because of the novel rather than familiar stimuli type. The weak modulation of recognition memory processes by encoding time may arise from the lack of recruitment of memory processes in a perceptual task, as well as from the insufficient statistical evidence due to the low number of participants. Consequently, we cannot rule out that more practice will promote recognition memory processes due to a longer total exposure as previously shown in memory tasks. Additional studies with greater practice and number of participants should be carried out to evaluate the development of a recognition memory during discrimination practice. In summary, discrimination practice enhances *familiarity* with both brief and long encoding times but long encoding times specifically enhances *recall*. Thus, the behavioral judgments in the perceptual task are weakly associated to the overall awareness of prior experience with the stimuli and strongly associated with the detailed memory for the viewed stimuli for long encoding times. Finally, the recognition memory processes are engaged and modulated in the perceptual task, although there are no specific instructions on memorizing stimuli.

While the results presented here further our understanding of ODL and the development of perceptual and recognition memory processes, they also have several limitations. In the first place, we used a sequential matching task that encompasses the perception of the first image, followed by its encoding and storage in STM, the perception of the second image and its comparison with the contents of STM. Because a fraction of the performance improvement reflects general processing abilities like encoding and storage in STM, to evaluate the possible specific contribution of STM we performed an additional experiment with six discrimination practice sessions where two 100 ms stimuli separated by 150 ms were presented, without a perceptual mask. The results showed an overall performance improvement after the six sessions that was fully transferred to the sequential matching task described in this work (data not shown), suggesting that for the results presented in this work, the contribution of coding and storage in STM is not significant. In addition, we previously showed that over 50% of the performance improvement during the practice sessions is not transferred to a different category of complex multiexemplar stimuli and is thus specific to the stimuli category (Montefusco-Siegmund et al., 2018). Taken together, the results presented here further our understanding the modulation of perceptual and recognition memory process by discrimination practice and encoding time during ODL.

## Conclusion

In the present work, we provide evidence that ODL of complex multiexemplar stimuli is primarily shaped by the extent of discrimination practice for stimuli durations equal or greater than 100 ms, and that long encoding times result in a greater discrimination performance regardless of the amount of practice. Second, and of greater relevance, ODL is similar for 100 and 1,000 ms encoding times, suggesting that the early stages of visual processing such as image decoding and coarse feature extraction are sufficient for ODL. These results also reveal a dissociation between the type of information required for ODL and for discrimination performance in a sequential matching task.

Furthermore, the *perceive same* and *know* processes of perception are enhanced by discrimination practice and both are associated with discrimination performance for brief and long encoding times, suggesting that practice shapes performance through the modulation of state and strength processes. In turn, the *familiarity* is enhanced by discrimination practice and is weakly associated with discrimination practice for brief and long stimuli and to performance for long stimuli, indicating the acquisition of a general awareness of previous occurrences for the viewed stimuli. Moreover, the *recall* process, weakly shaped by encoding time, is associated with performance for long encoding times, suggesting that long exposures in a perceptual task foster conscious recollection with long encoding time, involving fine feature extraction. These findings contribute to revealing a role of the early stages of visual processing in ODL and provide evidence on the progression of the perception and recognition memory processes during discrimination practice and its relationship with ODL.

## Data Availability Statement

The datasets presented in this study are available in http://dx.doi.org/10.17632/ctz4vgk9d7.1 and Supplementary Material.

## Ethics Statement

The studies involving human participants were reviewed and approved by Comité Ético Científico, Universidad de Talca. The patients/participants provided their written informed consent to participate in this study.

## Author Contributions

All authors listed have made a substantial contribution to the work. MQ performed the experiments. DG designed and programmed the experiments. RM-S analyzed the data and wrote the manuscript. MA designed and supervised the experiments, analyzed the data, and wrote the manuscript.

## Conflict of Interest

The authors declare that the research was conducted in the absence of any commercial or financial relationships that could be construed as a potential conflict of interest.

## Footnotes

1 www.palamedestoolbox.org
