# Supplementary material for: Early Visual Processing and Perception Processes in Object Discrimination Learning
Source: Front Neurosci. 2021 Jan 28;15:617824. doi: 10.3389/fnins.2021.617824 (PMC7876415; doi:10.3389/fnins.2021.617824)
Supplement: Supplementary file 1 [file Data_Sheet_1.PDF]

Supplementary Material

Figure 2. Discrimination learning of complex images for brief and long stimuli.

The lines in Figure 2 were calculated by fitting the mean data to a quadratic equation with Matlab.

dPrime/Brief Fitting

```
f = fit(xAxis',stat.dprimeBriefSessions.mean','poly2')
Linear model Poly2:  f(x) = p1*x^2 + p2*x + p3

Coefficients (with 95% confidence bounds):
    p1 =   -0.02833  (-0.03269, -0.02397)
    p2 =     0.5779  (0.5292, 0.6266)
    p3 =      1.264  (1.147, 1.381)
```

dPrime/Long Fitting

```
f2 = fit(xAxis',stat.dprimeLongSessions.mean','poly2')
Linear model Poly2: f2(x) = p1*x^2 + p2*x + p3

Coefficients (with 95% confidence bounds):
    p1 =   -0.03458  (-0.09577, 0.0266)
    p2 =     0.6762  (-0.007507, 1.36)
    p3 =      1.963  (0.326, 3.601)
```
